# Putative Bronchopulmonary Flagellated Protozoa in Immunosuppressed Patients

**DOI:** 10.1155/2014/912346

**Published:** 2014-04-03

**Authors:** Ali Ahmet Kilimcioglu, Yavuz Havlucu, Nogay Girginkardesler, Pınar Çelik, Kor Yereli, Ahmet Özbilgin

**Affiliations:** ^1^Department of Parasitology, Faculty of Medicine, Celal Bayar University, 45030 Manisa, Turkey; ^2^Department of Chest Disease, Faculty of Medicine, Celal Bayar University, 45030 Manisa, Turkey

## Abstract

Flagellated protozoa that cause bronchopulmonary symptoms in humans are commonly neglected. These protozoal forms which were presumed to be “flagellated protozoa” have been previously identified in immunosuppressed patients in a number of studies, but have not been certainly classified so far. Since no human cases of bronchopulmonary flagellated protozoa were reported from Turkey, we aimed to investigate these putative protozoa in immunosuppressed patients who are particularly at risk of infectious diseases. Bronchoalveolar lavage fluid samples of 110 immunosuppressed adult patients who were admitted to the Department of Chest Diseases, Hafsa Sultan Hospital of Celal Bayar University, Manisa, Turkey, were examined in terms of parasites by light microscopy. Flagellated protozoal forms were detected in nine (8.2%) of 110 cases. Metronidazole (500 mg *b.i.d.* for 30 days) was given to all positive cases and a second bronchoscopy was performed at the end of the treatment, which revealed no parasites. In conclusion, immunosuppressed patients with bronchopulmonary symptoms should attentively be examined with regard to flagellated protozoa which can easily be misidentified as epithelial cells.

## 1. Introduction

Protozoa such as Microsporidia,* Cryptosporidium, Entamoeba histolytica,* and* Leishmania* which are single-cell eukaryotes, are amongst the microorganisms that rarely cause infections in the respiratory system. Severity of these infections can vary according to the immune status, which is altered in AIDS, organ transplantation, cancer, and corticotherapy [1].* Lophomonas blattarum* is another example of a single-cell eukaryote that occasionally appears to infect humans. Only a few articles reported that* Lophomonas blattarum* and other flagellated protozoa caused bronchopulmonary infections in humans [2–6]. No human cases of bronchopulmonary flagellated protozoa were reported from Turkey, but only a few cases were diagnosed as* L. blattarum* by Levent Doğancı (Bayındır Hospital, Turkey, personal communication).

Ribas et al. reported that, in the case of pulmonary infections in immunosuppressed patients, examining bronchial secretions including bronchoalveolar lavage fluid (BALf) would be more useful to detect certain microorganisms [7]. It has been argued that it was difficult to distinguish protozoal forms from epithelial cells as the morphological features are quite similar. In addition to the difficulty in differentiating the emerging parasite,* L. blattarum*, and the other flagellated protozoa in bronchial secretions, bronchial epithelial cells could easily be misidentified as flagellated protozoa [8, 9].* Lophomonas blattarum* is a flagellated protozoon found in order Hypermastigida and suborderLophomonadina. It is accepted as an endocommensal in the intestine of cockroaches such as* Periplaneta americana* and* Blattella orientalis*.* L. blattarum* is approximately 20–60 *μ*m in length and has round to oval shape [10].

Mode of transmission of these flagellated protozoa still remains a mystery. The most frequent symptoms in humans are fever, cough, and sputum expectoration [3]. Radiology may reveal signs of pneumonia, bronchiectasis, pulmonary abscess, and pleural effusion. Successful treatment by metronidazole has been reported [1–3]. Many researchers concluded that microscopic examination of the respiratory secretions was the essential method for diagnosis of protozoal forms [3, 7, 9].

Even though bronchoscopy is an invasive method, parasitic examination of BALf was performed in immunosuppressed patients in whom fiberoptic bronchoscopy (FOB) was indicated for other pathologies. The aim of this study was to investigate bronchopulmonary flagellated protozoa in immunosuppressed patients.

## 2. Materials and Methods

BALf samples were obtained by FOB (Olympus, EVIS EXERA II CV-180, Tokyo, Japan) from 110 immunosuppressed adult patients who were admitted to the Department of Chest Diseases, Celal Bayar University, Manisa, Turkey, between 2011 and 2012. BALf collection was performed by wedging the tip of the bronchoscope into the nondependent lobes, especially middle lobe of the right lung and lingula of the left lung in each patient. The BALf collected lobe was determined by the images of the lesion with the greatest radiological abnormality. About 100 mL of sterile physiologic saline warmed to the body temperature was instilled in 20 mL aliquots. Gentle manual suction was applied to retrieve the saline. BALf was collected in sterilized containers and brought to the laboratory. Apart from these, additional invasive surgery was not applied to the patients.

**Figure 1:**
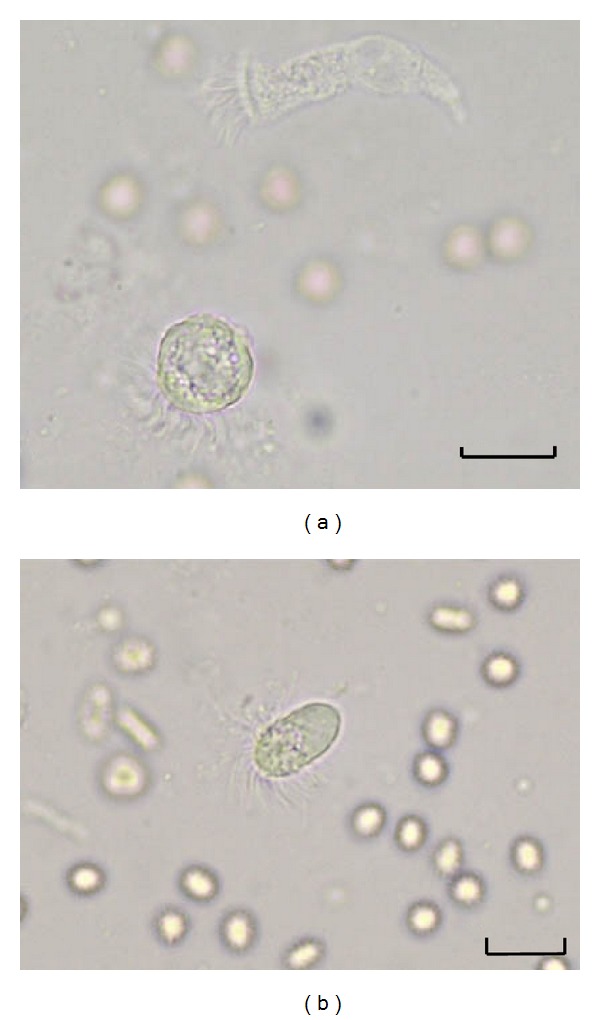
(a) A round shaped flagellated protozoon with granular cytoplasm and wavy, not combed flagella of different lengths without a terminal bar in BALf smear. At the top it is possible to observe a columnar ciliated epithelial cell (wet mount preparation ×400, case number 1). (b) An oval shaped flagellated protozoal form in BALf. Long flagella of variable length are inserted around the cytoplasm (wet mount preparation ×400, case number 2). Scale bar = 25 *μ*m.

BALf samples were examined directly and after centrifugation at 1000 ×g for 5 minutes under the light microscope (×400) within half an hour. A drop of BALf was put on the slide and covered with a coverslip for direct wet mount examination. This preparation was used primarily to observe the movements of cilia or flagella of putative protozoan and epithelial cells. Round or oval, motile trophozoites (20 to 60 *μ*m in length) with granular cytoplasm and wavy, not combed flagella of different lengths without a terminal bar were considered to be flagellated protozoa (Figures 1(a) and 1(b)). Round or columnar shaped cells with straight, combed, and uniform length cilia with rhythmic and synchronous movements were considered to be ciliated bronchial epithelial cells (Figure 1(a)).

**Figure 2:**
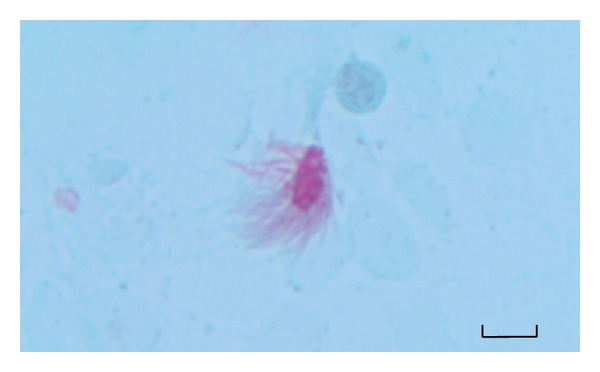
A putative bronchopulmonary flagellated protozoon in stained BALf smear (Wheatley's Trichrome stain ×1000). Long and irregular flagella are inserted around the cytoplasm. Scale bar = 25 *μ*m.

**Figure 3:**
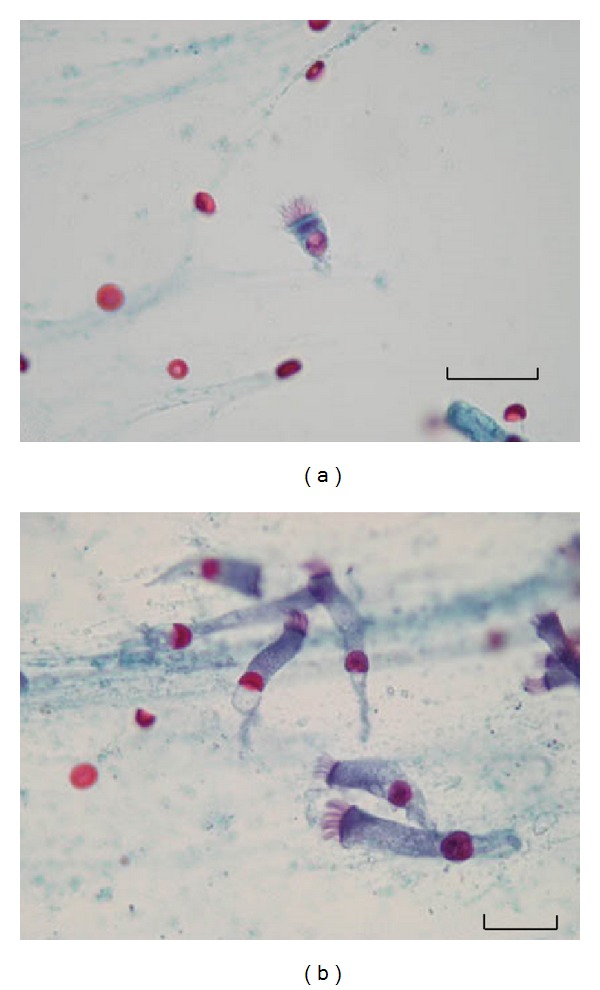
(a) A ciliated epithelial cell in BALf smear. Straight and combed cilia of the same length, inserted into a terminal bar, can be seen along one edge. A clear nucleus is also seen at the end of the cytoplasm (Wheatley's Trichrome stain ×1000). (b) A group of columnar epithelial cells in BALf smear. (Wheatley's Trichrome stain ×1000). Scale bar = 25 *μ*m.

BALf samples were stained using the trichrome technique of Wheatley [11]. Briefly, the BALf smeared slides were allowed to air dry for a few minutes following fixation in Schaudinn's fixative for at least 30 minutes. Then the staining process was performed as follows. First, slides were immersed in 70% alcohol for 5 minutes, followed by removal of mercuric chloride by 70% alcohol plus iodine for 1 minute. Iodine was then removed from the smear in two changes of 70% alcohol for 5 minutes of each and stained with trichrome stain for 10 minutes. For destaining, the slides were immersed in 90% alcohol plus acetic acid for 1 to 3 seconds and dipped several times in 100% alcohol as a rinsing step. Two changes of 100% alcohol for 3 minutes of each were used for dehydration followed by two changes of xylene for 5 to 10 minutes to complete the dehydration step. Finally, slides were covered with a coverslip and examined under light microscope using ×100 ocular piece. Protozoal forms and ciliated epithelial cells were distinguished based on the characteristic features as previously described by Ribas et al. [7]. A putative bronchopulmonary flagellated protozoon stained by Wheatley's trichrome is presented in Figure 2. Columnar cells having short, regular cilia and discernible terminal bar were considered to be ciliated bronchial epithelial cells (Figure 3).

Cases positive for protozoal forms were treated with metronidazole (500 mg* b.i.d.* for 30 days). Second FOB was carried out in positive cases as a follow-up control after treatment. A questionnaire was also given to positive cases.

Before the study, approval was obtained from the Ethics Committee of Faculty of Medicine, Celal Bayar University (approval number 118, dated May 18, 2011) and all patients were informed and written consents were taken.

## 3. Results

Flagellated protozoa were found in nine of 110 (8.2%) immunosuppressed patients.  Figure 1 presents protozoal forms of case number 1 and case number 2 in the BALf samples. Of these nine positive cases, eight were male, and one was female. It was found that most of the cases were farmers or factory laborers with low socioeconomic status. Sociodemographic, clinical, and laboratory findings of these cases were given in Table 1.

No flagellated protozoa were detected in BALf samples of all positive cases after treatment. In follow-up controls, it was observed that the initial pulmonary symptoms were considerably recovered in all cases.

## 4. Discussion

Only a few human cases have been reported in the world on bronchopulmonary infection caused by flagellated protozoa [7, 8]. However, presence of* L. blattarum, *which has similar morphology like other protozoal forms, has been reported in some studies [2–4, 12]. A detailed review based on extensive search of PubMed and Google Scholar about* L. blattarum* and bronchopulmonary protozoal infections has recently been published [13].

Ribas et al. [8] and Martínez-Girón et al. [9] have submitted “Letters to the Editor” claiming that some figures presented in the related manuscripts did not actually depict flagellated protozoa/*L. blattarum*, arguing that the bronchial epithelial cells could easily be misidentified as flagellated protozoa, which is in agreement with our experience in our series of 110 patients. A detailed table was reported which can be very useful to distinguish protozoal forms from ciliated epithelial cells [7]. Based especially on this table and the other literature, we were able to detect the protozoal forms and the bronchial epithelial cells in BALf samples though we could not differentiate* L. blattarum* from other protozoal forms. Thus, we defined all the parasitic forms we detected as flagellated protozoa. We observed nearly 1 or 2 bronchopulmonary flagellated protozoan cells per 10 microscopic fields (×400) while numerous epithelial cells were seen in each field in direct examination of BALf samples. Moreover, epithelial cells formed clusters in most of microscopic fields, so the protozoan cells could easily be overlooked. This paper highlights the need for molecular techniques to detect their presence and to differentiate the protozoal forms in respiratory secretions. Apart from these, culture studies we performed with Trypticase-Yeast-Maltose, Cystein-Peptone-Liver-Maltose, and Novy-Nicolle-McNeal media had unfortunately failed. We think successful cultivation of these parasites will enable us to describe detailed morphological features, to improve specific molecular techniques, and to develop novel treatment choices.

A cytological study [7] in which sputum smears of 106 immunocompromised patients (83 AIDS, 23 non-AIDS patients) were evaluated in terms of protozoal forms and compared with nonimmunocompromised group (control group, *n* = 85) with different respiratory disorders showed a greater number of protozoal forms in the sputa of patients with AIDS (86.7%) in comparison to the other two groups. Protozoal forms were found in 34.8% and 18.8% in non-AIDS immunocompromised patients and in control group, respectively, in that study. In a Letter to the Editor which reported an AIDS case [8], the authors stated that a multiflagellated protozoan cell was found in the aspiration fluid. Because FOB is an invasive method, healthy control group was not included in the present study, so it is unclear whether the protozoa observed in immunosuppressed cases are more common than healthy controls.

In a study in which the relationship between protozoa and asthma was evaluated [6], data supported the hypothesis that protozoa were more prevalent in patients diagnosed with asthma than in comparable controls. That study also investigated the relationship between presence of protozoa in sputum and dampness in the house and the data did not support the hypothesis that an increased prevalence of protozoa in sputum was associated with living in damp houses. However, as all of our positive cases were from low socioeconomic background, it was considered that the home in which the patients lived and hygienic conditions might contribute to the occurrence of the parasites. Furthermore, it was thought that the presence of cockroaches in abundance in such houses could play a crucial role for infection [1], but in our cases such a risk was not encountered.

In our study, the cases infected with flagellated protozoa were treated with metronidazole and after the treatment a second FOB and chest radiography were performed for follow-up control. One of the common points in the literature was to use metronidazole in the treatment [4, 8]. However, the dosage and the length of the treatment period were different from one case to another. We considered that the considerable recovery of symptoms and improvements in radiological findings of our cases in the follow-up controls supported the accuracy of our diagnosis and the treatment choice as well.

We conclude that immunosuppressed patients with bronchopulmonary symptoms should attentively be examined with regard to flagellated protozoa which can easily be misidentified as epithelial cells.

## Figures and Tables

**Table 1:** Sociodemographic, clinical, and laboratory findings of nine cases detected with flagellated protozoal forms.

	Case 1	Case 2	Case 3	Case 4	Case 5	Case 6	Case 7	Case 8	Case 9
Gender	Male	Male	Male	Male	Female	Male	Male	Male	Male
Age	48	60	56	76	71	49	74	78	67
Occupation	Worker	Farmer	Worker	Farmer	Housewife	Worker	Farmer	Farmer	Retired
Socioeconomic status	Low	Low	Low	Low	Low	Low	Low	Low	Low
Presence of cockroaches in the house	Yes	No	Yes	No	Yes	Yes	No	No	Yes
Symptom	Weakness	Weight loss	Dyspnea	Weight loss	Cough	Dyspnea	Dyspnea	Dyspnea	Weakness
Comorbidity	Diabetes mellitus	Psoriasis	Lung cancer	Kaposi sarcoma	COPD	COPD	COPD, hypertension	Alzheimer, nasopharyngeal carcinoma	Psoriasis
Radiological findings	Reticular infiltration	Reticular infiltration and bronchiectasis	Alveolar opacities in right upper lung	Reticular infiltration	Reticular infiltration	Lung abscess and pyothorax	Lobar infiltration	Multilobar infiltration and atelectasis	Reticular infiltration
White blood cell (×10^9^)	7.7	10.8	9.6	6.5	14.2	14.7	11.6	27.0	13.7
Neutrophil (%)	65.4	58.5	63.7	45.7	69.3	64.1	52.4	71.4	56.9
Lymphocyte (%)	25.1	30.2	22.5	38.6	20.1	22.6	21.6	22.6	27.2
Eosinophil (%)	2.4	4.7	4.1	7.1	3.3	1.4	11.3	2.8	7.4
Erythrocyte sedimentation rate	65	88	69	81	46	94	53	104	59
Presence of malignancy	No	No	Yes	Yes	No	Yes	Yes	Yes	No
Usage of corticosteroid	Yes	No	No	Yes	No	Yes	No	Yes	No
Usage of anti-TNF-*α* drugs	No	Yes	No	No	No	No	No	No	Yes
Duration of metronidazole treatment (days)	30	30	30	30	30	30	30	30	30
Flagellated protozoa after treatment	Eradicated	Eradicated	Eradicated	Eradicated	Eradicated	Eradicated	Eradicated	Eradicated	Eradicated

COPD: Chronic obstructive pulmonary disease.
